# Integrated multi-omic profiling reveals macrophage-driven prognostic signatures in clear cell renal cell carcinoma through machine learning optimization

**DOI:** 10.3389/fimmu.2025.1612262

**Published:** 2026-01-12

**Authors:** Wei Cao, Wenyuan Zhuang, Kai Xu

**Affiliations:** Department of Urology, The Third Affiliated Hospital of Nanjing Medical University, Changzhou, Jiangsu, China

**Keywords:** clear cell renal cell carcinoma (KIRC), tumor microenvironment (TME), tumor-associated macrophages (TAMs), single-cell RNA sequencing (scRNA-seq), high-dimensional weighted gene co-expression network analysis (hdWGCNA), machine learning, prognostic biomarkers

## Abstract

**Objective:**

Clear cell renal cell carcinoma (KIRC) is the most prevalent and aggressive form of kidney cancer, with limited survival despite advances in combination immunotherapy. Tumor-associated macrophages (TAMs) critically shape the tumor microenvironment (TME) and influence treatment resistance. We aimed to delineate TAM heterogeneity, identify prognostic macrophage signatures, and characterize the immune-metabolic programs underpinning KIRC progression.

**Methods:**

We integrated single-cell RNA sequencing (scRNA-seq) data from ten KIRC tumors with high-dimensional weighted gene co-expression network analysis (hdWGCNA) and twenty machine-learning models. Five macrophage subpopulations were defined by canonical markers and validated spatially. A macrophage-centric prognostic signature was trained using a random survival forest model and validated in another independent cohort. We further interrogated mutational landscapes, immune-stromal infiltration (xCell), pathway activation (ssGSEA), and clinical correlations.

**Results:**

scRNA-seq identified five transcriptionally distinct macrophage (Mac) subsets, including three lipid-associated Mac populations (LA-Mac: ALOX5AP+LA-Mac, HERPUD1+LA-Mac, and PRDX1+LA-Mac), an FCN1+inflammatory Mac subset (FCN1+Inflam-Mac), and an oxidative phosphorylation-enriched subset (OxP-Mac), distinct from canonical M1/M2 signatures. hdWGCNA revealed ten co-expression modules, among which the Mac-M2 module demonstrated the highest macrophage specificity and was preferentially enriched in tumor tissues. Based on seven hub genes from the Mac-M2 module, RSF model was constructed, achieving robust prognostic performance and effectively stratifying patients into high- and low-risk groups (log-rank *p* < 0.0001) in both the TCGA and CPTAC cohorts. Deconvolution analysis and gene set scoring of macrophage subtypes consistently identified PRDX1+LA-Mac as the predominant and prognostically relevant pathological subtype enriched in high-risk patients across both TCGA and CPTAC cohorts. Moreover, high-risk patients in TCGA exhibited elevated tumor mutation burden, increased pro-inflammatory M1 macrophages, Th2 polarization, metabolic dysregulation, and enhanced EMT signatures, all correlating with poorer survival.

**Conclusion:**

This multi-omics study illuminates the transcriptional and functional heterogeneity of TAMs in KIRC and establishes a macrophage-derived prognostic signature with translational potential. Our findings underscore the dual roles of macrophage polarization in mediating immune suppression and metabolic adaptation, offering novel targets for clinical diagnosis and treatment of KIRC.

## 1 Introduction

Clear cell renal cell carcinoma (KIRC) represents the most prevalent and aggressive subtype of renal cell carcinoma, accounting for approximately 75-80% of all kidney cancers and ranking as the third most common urological malignancy worldwide (1). Although the treatment landscape has shifted toward combination immunotherapy regimens—specifically, immune checkpoint inhibitors combined with tyrosine kinase inhibitors, clinical outcomes remain suboptimal. Approximately 35% of KIRC cases relapse, and the 5-year overall survival rate ranges from 44% to 69%, in contrast to 82–92% for papillary tumors and 78–92% for chromophobe tumors (2–4). Accumulating evidence indicates that the complex tumor microenvironment (TME) plays a pivotal role in mediating treatment resistance and influencing clinical outcomes, making it a key focus for therapeutic intervention in cancers including KIRC (5, 6). Bidirectional crosstalk between malignant cells and TME components establishes self-reinforcing feedback loops that promote therapeutic evasion (7, 8).

Macrophages are pivotal for maintaining tissue homeostasis, orchestrating immune responses, and facilitating tissue repair, while under certain conditions they can contribute to disease pathogenesis (9). Tumor-associated macrophages (TAMs), among the most abundant and functionally diverse immune populations within the tumor microenvironment (TME), play critical roles in tumor progression, metastasis and drug resistance (10). Their phenotypic diversity has been observed across multiple cancer types, with specific TAM subtypes associated with poor prognosis (11–13). Cancer cells secrete factors such as CSF-1, CCL2, and IL-4 that recruit and polarize macrophages toward pro-tumorigenic M2 phenotypes. In turn, M2-like TAMs facilitate tumor growth through immunosuppression, angiogenesis, and metabolic reprogramming—creating a self-sustaining cycle that accelerates disease progression and therapeutic resistance (14–18). This bidirectional interaction positions TAMs as both mechanistic drivers of disease and promising therapeutic targets. Strategies to target TAMs—either through depletion or functional reprogramming—have shown promise in preclinical and clinical studies (19, 20). For example, CSF1R inhibitors (pexidartinib, PLX3397) have demonstrated clinical activity and are under investigation in phase II trials for pancreatic adenocarcinoma and glioblastoma (20). Moreover, functional reprogramming using CD40 agonists, TLR agonists, and PI3Kγ inhibitors has successfully redirected M2-like TAMs toward M1-like anti-tumor phenotypes in melanoma, pancreatic cancer, and breast cancer (21–23). While significant progress has been made in understanding TAM biology in several cancer types, growing evidence highlights the remarkable heterogeneity of TAMs—not only in their phenotypic polarization (e.g., M1 *vs*. M2) but also in their transcriptional programs, functional roles, and spatial distributions across distinct tumor contexts (24, 25). These tumor-specific differences underscore the need to study TAMs within each cancer type individually. However, in KIRC, TAM-related studies remain limited and fragmented, with little understanding of their transcriptomic modular architecture, subtype diversity, and functional roles within the tumor microenvironment.

To address these critical gaps, we conducted a comprehensive multi-omics investigation integrating single-cell RNA sequencing (scRNA-seq), high-dimensional weighted gene co-expression network analysis (hdWGCNA), and machine learning approaches to systematically dissect the molecular landscape of TAMs in KIRC. Our study aims to: (1) define the transcriptional heterogeneity and functional specialization of macrophage subpopulations within KIRC tumors; (2) elucidate the gene co-expression networks and regulatory modules governing macrophage polarization and function; (3) develop and validate a robust macrophage-centric prognostic signature for risk stratification; and (4) characterize the mutation, immune and transcriptome features associated with risk stratification. Through this integrative framework, we provide translational evidence supporting macrophage-targeted therapeutic strategies and identify novel biomarkers for prognosis prediction and treatment in KIRC.

## 2 Materials and methods

### 2.1 Data collection

The KIRC bulk RNA-seq dataset was available data from The Cancer Genome Atlas (TCGA, https://portal.gdc.cancer.gov/) and Clinical Proteomic Tumor Analysis Consortium (CPTAC, https://pdc.cancer.gov/pdc/browse) KIRC cohorts (26, 27). The previously published KIRC scRNA-seq data from normal kidney, tumor-adjacent, and tumor tissues was downloaded from CELLxGENE database (https://cellxgene.cziscience.com/collections/f7cecffa-00b4-4560-a29a-8ad626b8ee08) (28). Only patients diagnosed with clear cell renal cell carcinoma (ccRCC), also referred to as KIRC, were included in this study. All data were retrieved and analyzed using established bioinformatics pipelines; no novel algorithms or computational methods were developed in this study.

### 2.2 Single-cell sequencing analysis

The scRNA-seq data were analyzed using the Seurat package in R (28). Cells were filtered based on quality control metrics, including the number of detected genes, total gene expression counts and the percentage of mitochondrial gene expression. Specifically, cells with less than 200 expressed genes, counts less than 1000 or more than 10000, or percentage of mitochondrial gene expression larger than 20%, were removed. Data were normalized using log normalization, and variable features were identified using the FindVariableFeatures function. Principal component analysis (PCA) was performed for dimensionality reduction[9], and the first 20 principal components were used for Uniform Manifold Approximation and Projection (UMAP) visualization. Cell clusters were identified using the FindClusters function with a resolution parameter of 0.5. Cell types were identified based on canonical gene markers as follows: CD3D, CD3E, CD4, LTB, IL7R, and MAL for CD4+ T cells; CD3D, CD8A, CD8B, GZMK, and NKG7 for CD8+ T cells; GNLY, GZMB, NKG7, KLRD1, KLRF1, and CD247 for natural killer (NK) cells; MS4A1, CD79A, CD79B, and CD19 for B cells; IGKC, IGHG1, JCHAIN, MZB1, and TNFRSF17 for plasma cells; LYZ, MRC1, CD68, CD163, and C1QC for macrophages; PLD4, ITM2C, LILRA4, IRF7, and IRF8 for plasmacytoid dendritic cells (pDCs); CD1C, CD86, HLA-DRA, and HLA-DRB5 for conventional dendritic cells (cDCs); TPSAB1 and TPSB2 for mast cells; PLVAP and TIMP3 for endothelial cells; ACTA2 and TAGLN for fibroblasts; GPX3 and GATM for proximal tubule (PT) epithelial cells; DEFB1 and UMOD for non-PT epithelial cells; and CA9 for renal cell carcinoma (RCC) cells.

### 2.3 Stacked bar plots and dot plots

The proportions of different cell types in normal kidney, tumor-adjacent, and tumor tissues were visualized using stacked bar plots. The expression levels of cell-type-specific markers were displayed using dot plots, where the size of each dot represented the percentage of cells expressing the marker, and the color represented the average expression level.

### 2.4 Enrichment analysis of cell types across conditions

To evaluate the overrepresentation of specific macrophage subtypes across different tissue or clinical conditions, we calculated the odds ratio (OR) and significance levels using Fisher’s exact test based on a 2×2 contingency table framework (29). For each combination of cell type and condition, we computed the number of cells belonging to a specific cell type within a given condition, compared to the number of other cells across all other combinations. The following categories were considered: (1) target cell type in target condition, (2) target cell type in all other conditions, (3) all other cell types in target condition, and (4) all other cell types in other conditions. The contingency table was analyzed using the fisher.test() function in R. P-values were adjusted for multiple comparisons using the Benjamini-Hochberg (BH) method. Full code is available in github (xxx).

### 2.5 Macrophage subpopulation analysis

Macrophage subpopulations were identified using unsupervised clustering of scRNA-seq data. Five transcriptionally distinct subsets were delineated and visualized using UMAP. Subtype annotation was performed based on enriched marker genes, integrating both functional characteristics and previously reported molecular signatures (29, 30). Based on this comprehensive analysis, we identified and annotated five distinct macrophage subsets: (1) ALOX5AP+ lipid-associated macrophages (ALOX5AP+LA-Mac), characterized by high expression of ALOX5AP, APOE, and APOC1, indicative of lipid metabolism and arachidonic acid processing; (2) HERPUD1+LA-Mac, marked by HERPUD1, APOE, and APOC1, suggesting endoplasmic reticulum stress response within lipid-metabolic macrophages; (3) PRDX1+LA-Mac, defined by PRDX1, APOE, and APOC1, representing antioxidant-responsive lipid-associated macrophages; (4) FCN1+ inflammatory macrophages (FCN1+Inflam-Mac), characterized by FCN1, IL1B, and ISG15 expression, indicative of pro-inflammatory activation; and (5) oxidative phosphorylation macrophages (OxP-Mac), marked by mitochondrial genes ATP5ME, MT-ND3, and MT-ND2, reflecting enhanced metabolic activity. Heatmaps were generated to display the z-score normalized expression of subtype-specific markers. The proportions of each macrophage subpopulation in different tissue contexts were analyzed. The functional polarization analysis was performed to assess M1/M2 polarization scores by the AddModuleScore function from Seurat package. The M1/M2 gene signatures were obtained from previously published research (31). Pseudotemporal trajectory analysis was used to reveal the dynamics of macrophage subpopulation differentiation by R package Monocle3 (31). The OxP-Mac was set to be starting point in trajectory analysis. The trajectory regulation genes were determined by the graph_test function from Monocle3 package with the threshold of adjusted p value < 1e-26 and Morans’ I > 0.25. Pathway enrichment analysis and gene set enrichment analysis (GSEA) were performed to identify key biological processes associated with each subpopulation.

### 2.6 High-dimensional weighted gene co-expression network analysis

The hdWGCNA package was used to identify gene modules from macrophages in scRNA-seq data (32). A soft threshold of 10 was chosen to construct the network, achieving the best scale-free topology. Key modules were defined as those specifically enriched in macrophages and highly expressed in tumor tissues. Volcano plots were generated to highlight the most significantly differentially expressed modules between tumor and control samples (including both tumor-adjacent and healthy tissues). Core genes were identified based on high module membership (kME) and differential expression between macrophages from tumor and control samples.

### 2.7 Prognostic model construction and validation

To assess the prognostic value of macrophage-related core genes, we constructed 20 prognostic models by combining different machine learning algorithms and parameter settings. These included Random Survival Forest (RSF), Elastic Net (Enet), Stepwise Cox, CoxBoost, Partial Least Squares Regression for Cox (plsRcox), Supervised Principal Components (SuperPC), Generalized Boosted Regression Modeling (GBM), and Survival Support Vector Machine (survival-SVM). Genes that overlapped between macrophage-related core genes and those associated with poor prognosis in the TCGA cohort were selected for model training. The TCGA-KIRC cohort was used as the training set, while the CPTAC-KIRC cohort served as the validation set. Prior to machine learning analysis, the gene expression data were log2-transformed and standardized (zero mean, unit variance) to minimize heterogeneity and ensure comparability across features. The RSF algorithm was selected for its optimal performance. Survival analysis, including Cox proportional hazards and Kaplan–Meier (KM) analyses, was conducted using the survival R package. For KM analysis, the optimal cutoff value was determined using the surv_cutpoint function from the survminer package, which stratified samples into high- and low-risk groups. Kaplan–Meier survival curves were visualized using the ggsurvplot function from survminer to compare survival outcomes between the high-risk and low-risk patients, as defined by the RSF model. A time-dependent receiver operating characteristic (ROC) curve was used to evaluate the prognostic performance of the RSF model. Feature importance analysis was performed to identify key prognostic genes.

### 2.8 Macrophage subtypes deconvolution analysis using MuSiC

To quantify the proportions of macrophage subpopulations in bulk transcriptomic datasets, we applied the MuSiC (Multi-subject Single Cell deconvolution) algorithm, which uses cross-subject single-cell RNA-seq references to infer cellular composition in bulk samples (33). We used macrophage subpopulations of single-cell RNA-seq data as the reference. MuSiC deconvolution was performed on the TCGA and CPTAC KIRC bulk RNA-seq datasets. Prior to deconvolution, bulk expression matrices were log-normalized, and only genes present in both bulk and single-cell datasets were retained. We executed the analysis with the music_prop function from the MuSiC R package (v1.0.0) using default settings. The output provided relative abundance estimates of each macrophage subtype in every bulk sample.

### 2.9 Mutation profile analysis

The mutation profile, including missense, silent, nonsense, frameshift/in-frame insertions and deletions, and uninterrupted mutations, was analyzed, excluding synonymous mutations. Tumor mutation burden (TMB) was calculated based on the total number of somatic mutations. Pathway enrichment analysis, focusing on known oncogenic or user-defined pathways, was performed using the pathways function. Differences in mutational profiles between KIRC risk groups were assessed using the mafCompare function. All analyses in this section were conducted using the maftools R package (34).

### 2.10 Immune microenvironment characterization

The tumor microenvironment was estimated using the xCell R package (35). Differences in macrophage polarization between high-risk and low-risk groups were assessed using the Wilcoxon rank-sum test. For Kaplan–Meier (KM) analysis of macrophage polarization scores, the optimal cutoff value was determined using the surv_cutpoint function from the survminer package.

### 2.11 Transcriptome analysis and pathway activation analysis

Differentially expressed genes (DEGs) between high-risk and low-risk patients were identified using R package limma with a threshold of |log2 fold change| > 1 and adjusted p-value < 0.05 (36). Gene Ontology (GO) enrichment analysis was performed on both upregulated and downregulated genes by R package clusterProfiler (37). Single-sample gene set enrichment analysis (ssGSEA) of the HALLMARK gene set was conducted to assess pathway activity in high-risk and low-risk tumors (38). The HALLMARK gene set was obtained from the Molecular Signatures Database (MSigDB, https://www.gsea-msigdb.org/gsea/msigdb/index.jsp). All statistical analyses were conducted using R version 4.4.3. A p-value < 0.05 and adjusted p-value < 0.05 were considered statistically significant.

## 3 Result

### 3.1 Single-cell profiling reveals immune cell heterogeneity and tissue-specific enrichment in KIRC

**Figure 1:**
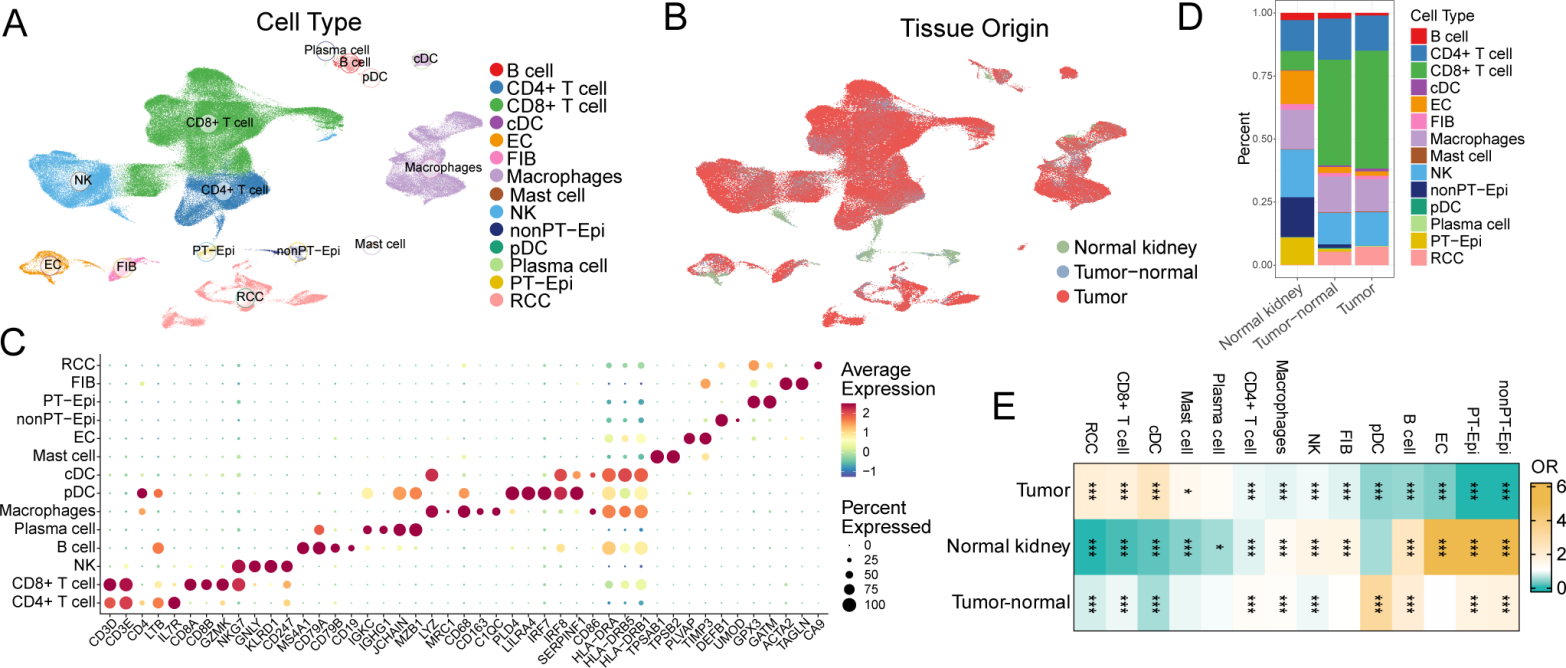
Single-cell atlas of immune and stromal cell heterogeneity in KIRC. **(A)** UMAP visualization of major cell populations across normal kidney, tumor-adjacent, and tumor tissues colored by cell types **(A)** and tissues **(B)**. **(C)** Dot plot of cell-type-specific marker expression. **(D)** Stacked bar plots showing proportional shifts of immune and stromal cell types. **(E)** Odds ratio (OR) analysis comparing cell-type enrichment across normal kidney, tumor-adjacent, and tumor tissues. Statistical significance was assessed using Fisher’s exact test. *p < 0.05, ***p < 0.001.

We first employed a KIRC scRNA-seq to explore the cellular heterogeneity across the tissue regions. After the quality control, normalization and cell annotation, a total of 187,290 cells were divided into 14 celltypes, including NK cells, B cells, plasma cells, classical dendritic cells (cDCs), plasma dendritic cells (pDCs), CD4+ T cells, CD8+ T cells, macrophages (Macs), mast cells, endothelial cells (ECs), fibroblasts (FIBs), non-proximal tubule epithelial cells (nonPT-Epi), proximal tubule epithelial cells (PT-Epi), and renal cell carcinoma (RCC) cells (**Figure 1A**). The tissue origins of cells were classified as normal kidney, tumor-adjacent, or tumor tissue (**Figure 1B**). The cell types were determined by the canonical marker genes, including CD3D, CD3E, CD4, LTB and IL7R for CD4+ T cells; CD3D, CD8A, CD8B, GZMK and NKG7 for CD8+ T cells; GNLY, NKG7, KLRD1 and CD247 for NK cells; MS4A1, CD79A, CD79B and CD19 for B cells; IGKC, IGHG1, JCHAIN and MZB1 for plasma cells; LYZ, MRC1, CD68, CD163 and C1QC for Macs; PLD4, LILRA4, IRF7, IRF8 and SERPINF1 for pDCs; CD86, HLA-DRA, HLA-DRB5 and HLA-DRB1 for cDCs; TPSAB1 and TPSB2 for mast cells; PLVAP and TIMP3 for ECs; DEFB1 and UMOD for nonPT-Epi; GPX3 and GATM for proximal tubule cells; ACTA2 and TAGLN for FIBs; and CA9 for RCCs (**Figure 1C**). The stacked bar chart displays the proportions of different cell types in normal kidney, tumor-adjacent, and tumor tissues, while the proportions of CD8+T cells and cDCs are significantly increased in tumor (**Figure 1D**). Moreover, the OR analysis quantifies the enrichment of different cell types in tumor tissues relative to normal tissues (**Figure 1E**). CD8+T cells, cDCs and RCCs are significantly enriched in tumor tissues (OR > 1), while the ECs and epithelial cells demonstrated the opposite pattern. Interestingly, Macs demonstrated the limited difference in OR (**Figure 1E**), suggesting that the functional alternation might be more important in KIRC progression. These findings revealed the significant heterogeneity of immune and stromal cells in KIRC.

### 3.2 Single-cell profiling delineates tumor-associated macrophage subpopulations with distinct functional states

**Figure 2:**
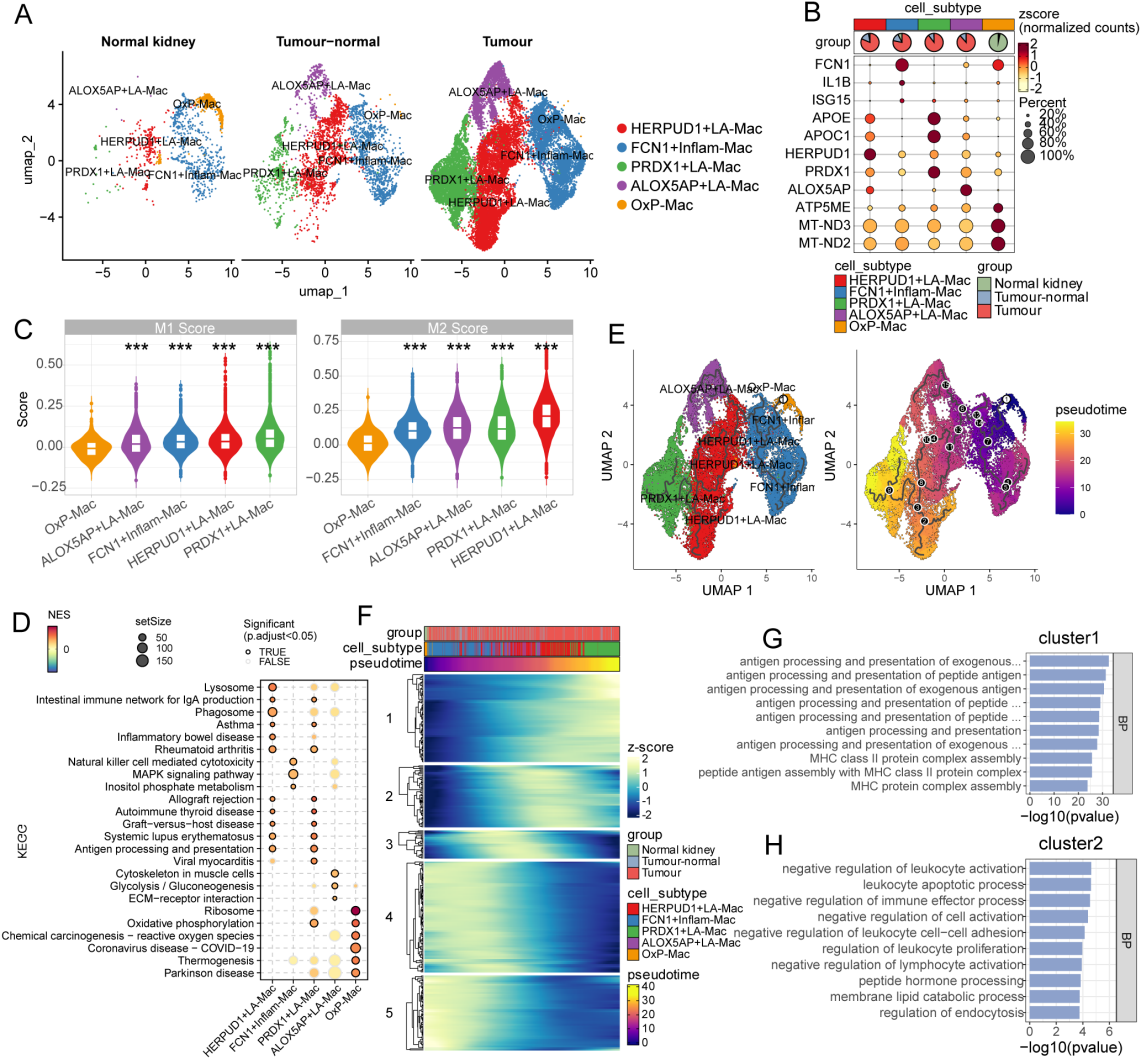
Tumor-associated macrophage subpopulations exhibit context-dependent functional specialization in KIRC. **(A)** UMAP visualization of five macrophage subpopulations: FCN1+Inflam-Mac, PRDX1+LA-Mac, HERPUD1+LA-Mac, ALOX5AP+LA-Mac, and OxP-Mac. **(B)** Dot plot of z-score-normalized expression for macrophage subtype-specific markers. The spatial distribution of macrophage subsets across normal kidney, tumor-adjacent, and tumor tissues were marker at the top panel. **(C)** Violin plot of M1/M2 polarization scores for each macrophage subset. **(D)** Dot plot of KEGG GSEA among the macrophage subtypes. Color indicates the normalized enrichment score (NES). **(E)** Pseudotime trajectory analysis illustrating differentiation dynamics colored by subtypes (left) and pseudotime (right). **(F)** Heatmap of representative regulation genes involved in macrophage subtypes differentiation, clustered into five distinct groups. **(G, H)** Functional enrichment of key biological processes (BP) for cluster1 and cluster2 genes which were enriched in HERPUD1+LA-Mac and PRDX1+LA-Mac, respectively. LA-Mac, lipid-associated macrophage; Inflam-Mac, inflammatory macrophage; OxP-Mac, oxidative phosphorylation macrophages. *** p<0.001.

We next sub clustered the Macs to explore the alternative activation in KIRC. According to the enriched marker genes integrated both functional characteristics and distinctive molecular signatures, macrophages (Mac) were identified five transcriptionally distinct subsets: (1) ALOX5AP+ lipid-associated macrophages (ALOX5AP+LA-Mac), characterized by high expression of ALOX5AP, APOE, and APOC1, indicative of lipid metabolism and arachidonic acid processing; (2) HERPUD1+LA-Mac, marked by HERPUD1, APOE, and APOC1, suggesting endoplasmic reticulum stress response within lipid-metabolic macrophages; (3) PRDX1+LA-Mac, defined by PRDX1, APOE, and APOC1, representing antioxidant-responsive lipid-associated macrophages; (4) FCN1+ inflammatory macrophages (FCN1+Inflam-Mac), characterized by FCN1, IL1B, and ISG15 expression, indicative of pro-inflammatory activation; and (5) oxidative phosphorylation macrophages (OxP-Mac), marked by mitochondrial genes ATP5ME, MT-ND3, and MT-ND2, reflecting enhanced metabolic activity (**Figures 2A, B**). Proportions of macrophage subpopulations shifted markedly across tissue contexts: OxP-Mac was enriched in normal tissues (up to 60%), while the other subtypes dominated tumor regions (**Figure 2B**). Subtype-specific markers validated by the z-score-normalized expression dotplot (**Figure 2B**). Functional polarization analysis showed these macrophage sub types were not canonical M1/M2 features, while tumor-enriched PRDX1+ and HERPUD1+LA-Mac exhibited mixed M1/M2 features (**Figure 2C**). Although the Mac substypes exhibited mix M1/M2 signatures, normal kidney enriched OxP-Mac demonstrated the lowest M1 as well as M2 scores compared to the other subtypes (**Figure 2C**), indicating a quiescent M0 state. Additionally, KEGG GSEA results highlighted an alternative functional activation among the Mac subtypes (**Figure 2D**). Specifically, HERPUD+ and PRDX1+LA-Macs demonstrated a similar phenotype characterized by hyper-inflammation and immune response while normal kidney enriched OxP-Macs were activated in ribosome and oxidative phyphorylation (**Figure 2D**).

We further employted pseudotime trajectory analysis to identify the development trajectory as well as regulation genes. Pseudotime trajectory analysis positioned OxP-Mac as an early-state population branching into tumor-specific subsets (ALOX5AP+, HERPUD1+ and PRDX1+LA-Mac), suggesting differentiation plasticity (**Figure 2E**). The regulation genes were divided into 5 clusters, among which cluster1 and cluster2 promoted this differentiation while cluster4 and cluster5 exhibited the opposite (**Figure 2F**). GO enrichment revealed that cluster1 regulation genes were functionally enriched in antigen presentation (MHC class II assembly, peptide antigen processing) (**Figure 2G**), while cluster2 genes were involved in leukocyte apoptosis and immune suppression (**Figure 2H**). These findings underscore functional diversification of tumor-associated macrophages and their potential roles in KIRC progression.

### 3.3 hdWGCNA identifies macrophage-specific gene modules and hub genes

**Figure 3:**
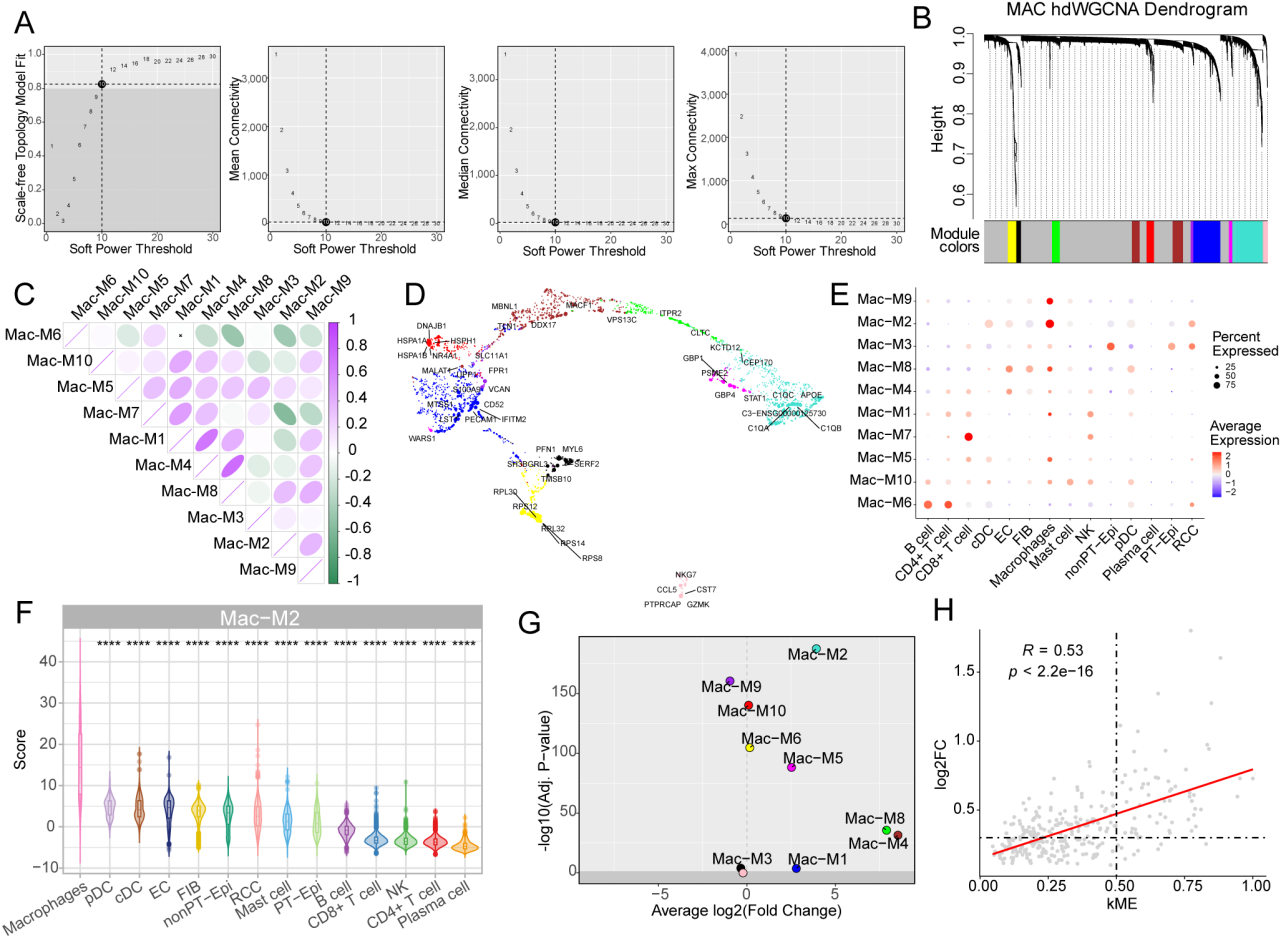
hdWGCNA reveals macrophage-specific gene co-expression modules and hub genes. **(A)** The average connectivity plot displays the scale-free topology fitting index and soft-thresholding ability. The optimal soft-threshold value is 10. **(B)** Hierarchical clustering dendrogram of gene modules. **(C)** Heatmap of module-to-module relationships based on the correlation between module eigengenes. **(D)** UMAP visualization of the hub gene network, with the top five hub genes labeled in each module. **(E)** Dot plot of module activity across different cell types, identified by the hdWGCNA algorithm. **(F)** Violin plots showing macrophage M2 module activity across different cell types. **(G)** Volcano plot of differential module expression. The x axis represents the tumor *vs* non-tumor log2 fold change (log2FC). The y axis represents the significance levels. **(H)** Dot plot of macrophage M2 genes, with the x-axis indicating eigengene-based connectivity (kME) and the y-axis showing log2FC values from differential expression analysis (tumor *vs*. non-tumor macrophages). The Spearman correlation coefficient and p-value are displayed in the top-left corner. ****p<0.0001.

To delineate macrophage-associated transcriptional networks, we applied high-dimensional weighted gene co-expression network analysis (hdWGCNA) to single-cell RNA-seq data. The optimal power of 10 was employed to construct a scale-free network which identified 10 gene co-expression modules (**Figures 3A, B**). The macrophage module 4 (Mac-M4) and Mac-M8 demonstrated a high correlation among these modules (**Figure 3C**). As **Figure 3D** shown, the top 5 genes were labelled in the UMAP. Among the 10 modules, Mac-M2 was highly enriched in macrophages compared to other cell types (p < 0.0001, **Figures 3E, F**), highlighting the genes of M2 were specifically expressed in macrophages. Moreover, M2 demonstrated the most differentially expressed modules with elevated log2 fold changes (log2FC > 3) and statistical significance (p < 1 × 10^-175^) (**Figure 3G**). Thus, Mac-M2 was selected for advanced analysis. To further clarify the hub genes in the Mac-M2 cluster, we employed two criteria: module membership (kME) and differential expression levels between tumor and normal macrophages (**Figure 3H**). Interestingly, these two values showed a strong correlation (r = 0.53, p < 2 × 10^-16^), suggesting that the associated genes may play a key role in macrophage differentiation (**Figure 3H**). Genes with kME > 0.5 and log2FC > 0.3 were selected for further analysis.

### 3.4 A macrophage-derived gene signature predicts clinical outcomes in KIRC patients

**Figure 4:**
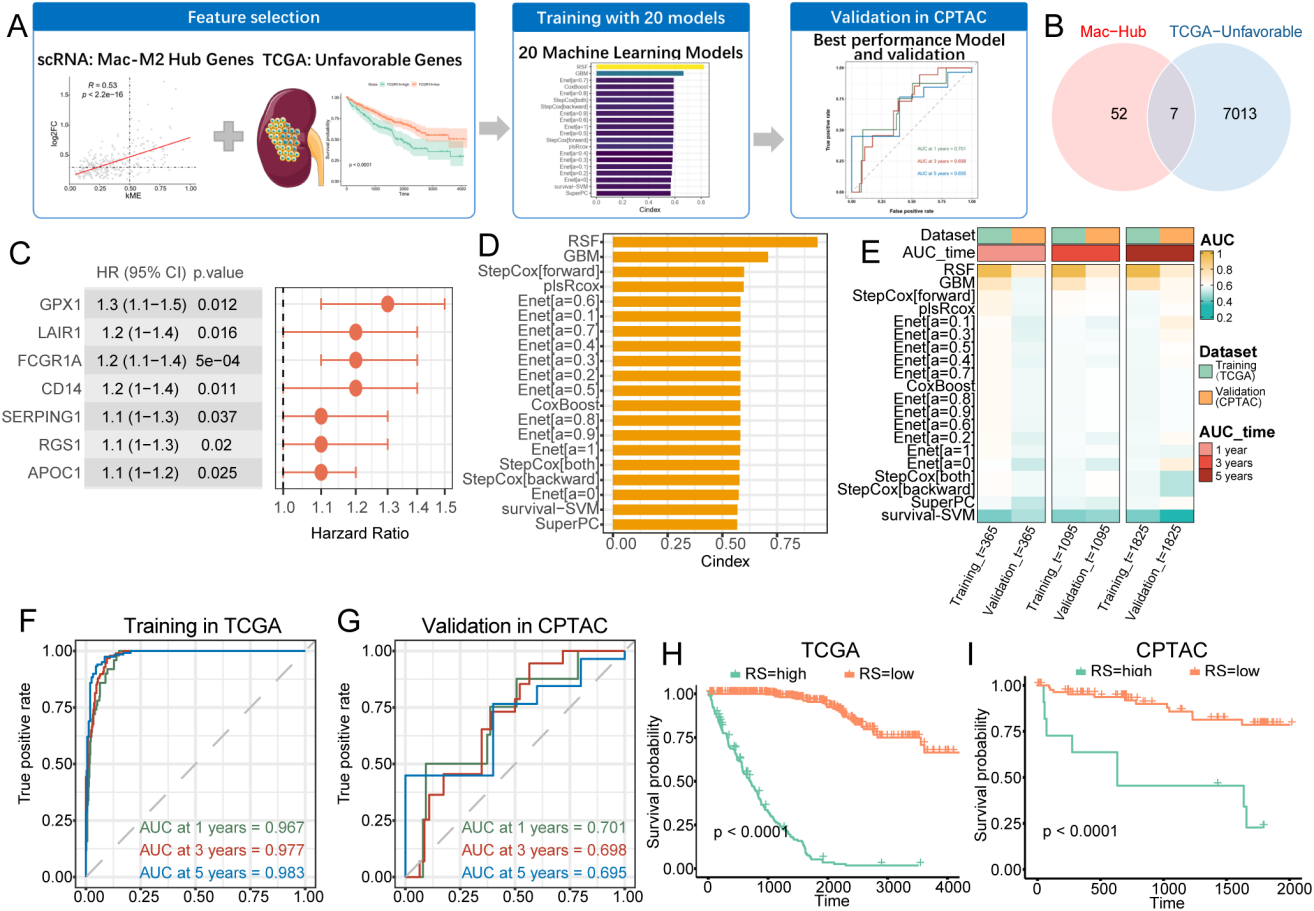
Macrophage-associated gene signature predicts survival and guides risk stratification in KIRC. **(A)** Machine Learning Workflow. **(B)** Venn plot of prognostic genes and macrophage-specific modules (Mac-Hub). **(C)** Forest plot of selected genes hazard ratio (HR). **(D)** Bar plot of C-index rankings of algorithms. **(E)** Heatmap plot of 20 survival algorithms in training and validation datasets with 1-, 3- and 5-years AUC values. **(F, G)** Time-ROC curves for training **(F)** and validation **(G)** cohorts, with AUC values at key timepoints. **(H, I)** Kaplan-Meier survival curves stratified by risk score in TCGA **(H)** and CPTAC **(I)** cohorts.

We constructed a prognostic model based on TCGA-KIRC data and validated it in the CPTAC-KIRC cohort to assess the predictive value of macrophage-associated hub genes (**Figures 4A–C**). Shared genes from the Mac-M2 module hub and TCGA-identified survival-related genes were used to build survival models (**Figures 4A–C**). Seven genes—GPX1, LAIR1, FCGR1A, CD14, SERPING1, RGS1, and APOC1—were selected during model training (**Figures 4B, C**). The TCGA-KIRC transcriptome served as the training set, while CPTAC-KIRC data were reserved for validation. Twenty machine learning models, generated through combinations of algorithms and parameter settings, were evaluated (**Figures 4A, D**). Among them, the Random Survival Forest (RSF) model demonstrated the best performance in the training cohort (C-index = 0.94), with 1-, 3-, and 5-year AUCs of 0.97, 0.98, and 0.98, respectively (**Figures 4D–F**). Although the CPTAC-KIRC validation AUCs were lower, they remained clinically meaningful at approximately 0.70 for 1-, 3-, and 5-year predictions (**Figure 4G**). This performance reduction may reflect heterogeneity between cohorts, particularly in median overall survival (TCGA: 1,188 days *vs*. CPTAC: 781 days, **Supplementary Figure S1A**) and survival status distributions (32.9% *vs*. 19.4%, **Supplementary Figure S1B**), although these differences did not reach statistical significance (log-rank p = 0.30, **Supplementary Figure S1C**). Patients were stratified into high- and low-risk groups based on the RSF risk scores; both groups showed significantly different survival outcomes in TCGA and CPTAC cohorts (log-rank p < 0.0001), confirming the model’s prognostic utility (**Figures 4H, I**).

### 3.5 PRDX1+LA-Mac and RSF feature genes are associated with unfavorable prognosis in KIRC patients

**Figure 5:**
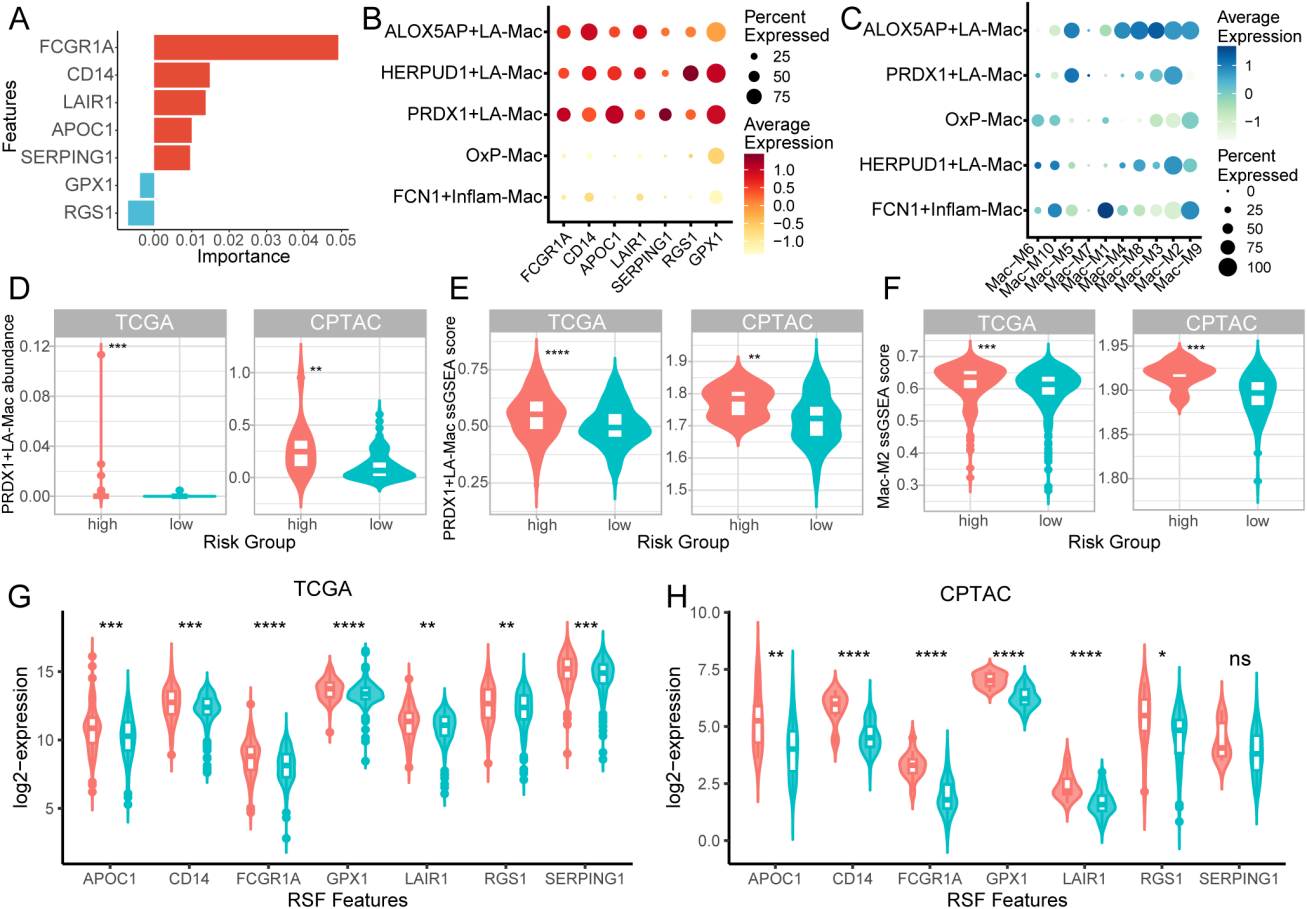
PRDX1+LA-Mac and RSF feature genes are enriched in high-risk KIRC patients and associated with poor prognosis. **(A)** Bar plot showing feature importance from the random survival forest (RSF) model. **(B)** Dot plot of RSF feature gene expression across macrophage subtypes identified by scRNA-seq. **(C)** Dot plot of macrophage module scores across macrophage subtypes in scRNA-seq data. **(D, E)** Violin plots showing the estimated abundance of macrophage subtypes by deconvolution **(D)** and ssGSEA scores **(E)** in TCGA (left) and CPTAC (right) cohorts. **(F)** Violin plot of ssGSEA scores for Mac-M2 hub genes in TCGA (left) and CPTAC (right) cohorts. **(G, H)** Violin plots showing expression of RSF feature genes in TCGA **(G)** and CPTAC **(H)** cohorts. *p < 0.05, **p < 0.01, ***p < 0.001, ****p < 0.0001.

To bridge our single-cell macrophage subtyping analysis with the prognostic model, we first examined the expression specificity of RSF feature genes across different macrophage subtypes identified in our scRNA-seq analysis. Among the genes contribution to RSF survival mode, FCGR1A had the highest variable importance, followed by CD14 and LAIR1 (**Figure 5A**). Notably, RSF feature genes were predominantly enriched in the ALOX5AP+, HERPUD1+, and PRDX1+LA-Mac subtypes (**Figure 5B**). Similarly, the Mac-M2 score exhibited the same pattern (**Figure 5C**), indicating that LA-Macs represent the cellular origin of Mac-M2 hub genes.

We then integrated single-cell–derived macrophage subtypes with bulk transcriptomic data from the TCGA and CPTAC cohorts using MuSiC deconvolution and ssGSEA scoring. MuSiC deconvolution revealed that PRDX1+LA-Macs were significantly enriched in high-risk patients compared to low-risk patients in both cohorts, whereas other macrophage subtypes displayed variable enrichment (**Figure 5D**; **Supplementary Figures S2A, B**). Consistently, ssGSEA of the top 30 subtype marker genes confirmed higher PRDX1+LA-Mac scores in high-risk patients (**Figure 5E**; **Supplementary Figures S2C, D**), and ssGSEA of the Mac-M2 hub genes showed the same trend (**Figure 5F**; **Supplementary Figures S2E, F**). Finally, individual RSF feature genes were significantly upregulated in high-risk patients in both cohorts—except for SERPING1 in CPTAC (**Figures 5G–H**). Collectively, these results implicate PRDX1+LA-Macs and Mac-M2 hub genes in driving unfavorable prognosis in KIRC patients.

### 3.6 TCGA data analysis reveals differences in mutation profiles between high-risk and low-risk KIRC patient groups

**Figure 6:**
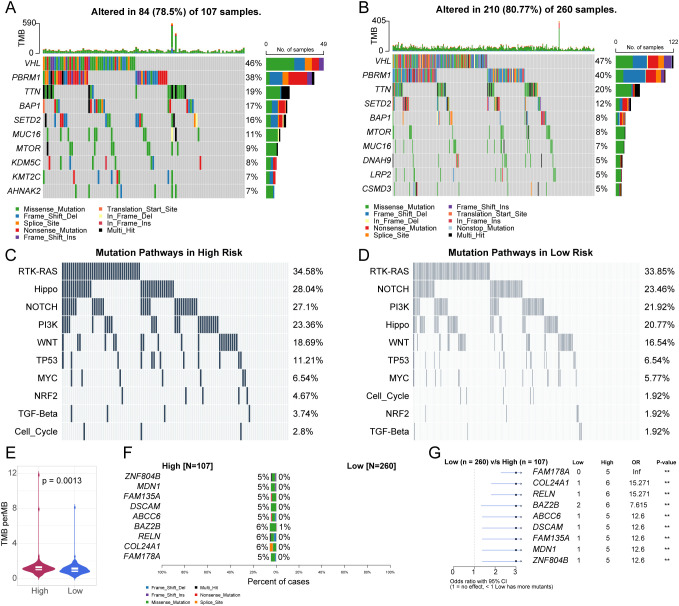
Comparison of Mutation Profiles between High-Risk and Low-Risk Groups of KIRC. **(A, B)** Waterfall plots displaying somatic mutations in KIRC high-risk **(A)** and log-risk groups **(B)**. **(C, D)** Waterfall plots illustrating oncogenic signaling pathways in KIRC high-risk **(C)** and low-risk groups **(D)**. **(E)** Violin plot comparing tumor mutational burden (TMB) between high-risk and low-risk groups. **(F)** Co-bar plot showing differentially mutated genes between high-risk and low-risk groups. **(G)** Forest plot of differentially mutated genes between high-risk and low-risk groups.

To further characterize molecular distinctions between high- and low-risk patient groups, we analyzed the mutation profile of the TCGA-KIRC cohort. In the high-risk group, 78.5% (84/107) of samples harbored at least one driver mutation; the most frequently mutated genes were VHL (46%), PBRM1 (38%), TTN (19%), and BAP1 (17%). Missense mutations and frameshift deletions predominated (**Figure 6A**). In the low-risk group, 80.8% (210/260) of samples carried driver mutations, with VHL (47%) and PBRM1 (40%) slightly more frequent; frameshift insertions were also more common (**Figure 6B**). Pathway enrichment analysis showed that mutations in the RTK-RAS (34.6%), Hippo (28.0%), and NOTCH (27.1%) pathways were significantly enriched in the high-risk group, whereas RTK-RAS (33.8%), NOTCH (23.5%), and PI3K (21.9%) pathways dominated in the low-risk group (**Figures 6C, D**). High-risk patients exhibited a significantly elevated tumor mutation burden (TMB) compared to low-risk patients (**Figure 6E**). Moreover, mutation frequencies of ZNF804B and FAM178A (5–6%) were significantly higher in high-risk patients, with odds ratios >3 (**Figures 6F, G**). These findings suggest that elevated TMB and specific gene mutations underlie the poorer prognosis observed in high-risk KIRC patients.

### 3.7 Macrophage-related features stratify survival outcomes in KIRC patients

**Figure 7:**
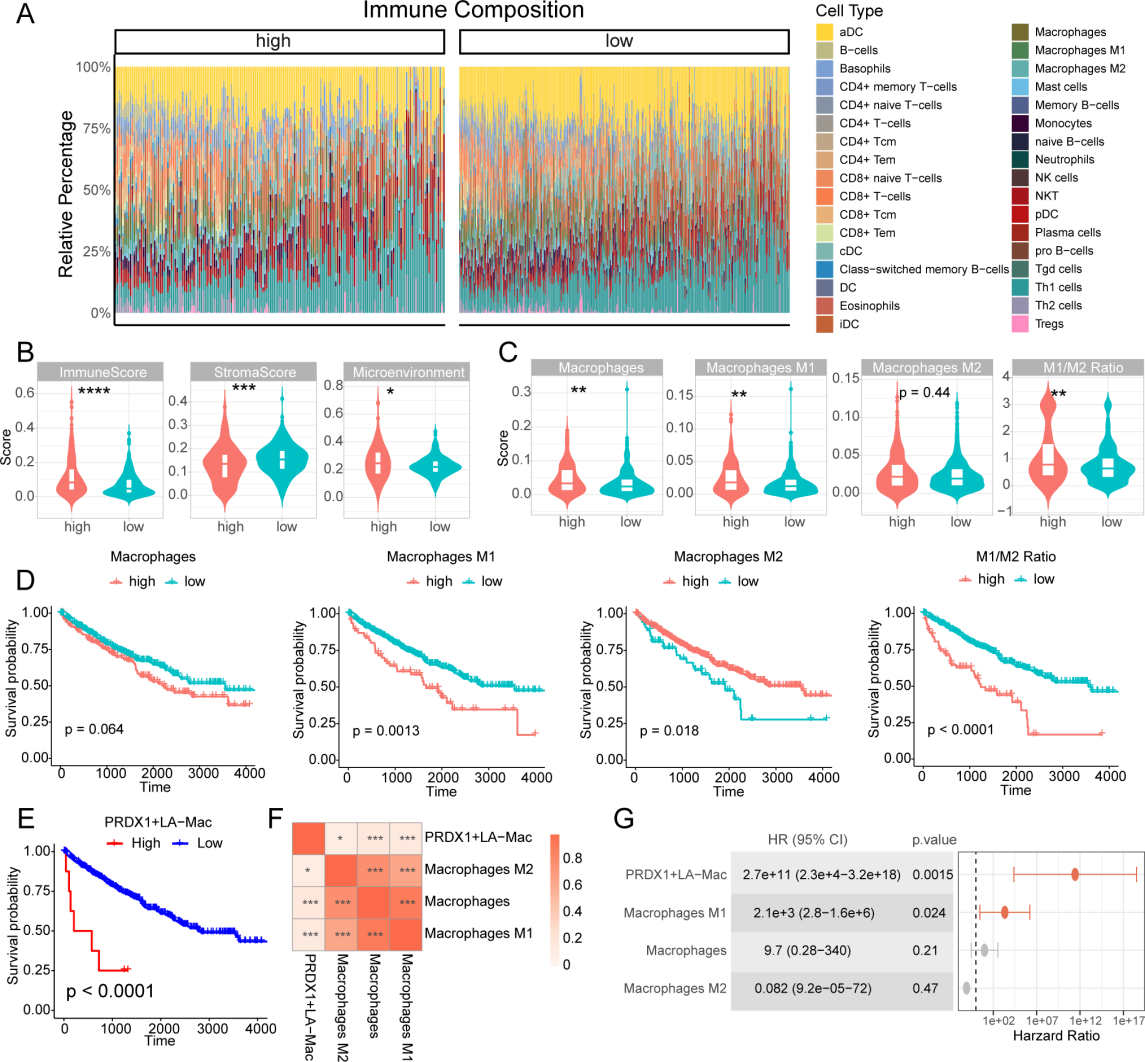
Immune microenvironment profiling links macrophage polarization to survival and immune evasion in KIRC. **(A)** Bar plot of tumor immune composition in high- and low-risk group patients of KIRC. **(B)** Violin plot comparing immune score, stromal score, and microenvironment score between high- and low-risk group patients of KIRC. **(C)** Violin plot of macrophage-related scores, including overall macrophage, M1, M2 and M1/M2 ratio, between high- and low-risk group patients of KIRC. **(D)** Kaplan-Meier (KM) plot for macrophage-related scores. **(E)** Kaplan–Meier survival analysis demonstrating the prognostic significance of the PRDX1^+^LA-Mac subtype in the TCGA-KIRC cohort. **(F)** Spearman correlation heatmap showing the association between PRDX1^+^LA-Mac abundance and classical macrophage subsets estimated by xCell (M1, M2, and total macrophages). **(G)** Forest plot illustrating the hazard ratios (HRs) of PRDX1^+^LA-Mac and classical macrophage populations derived from univariate Cox regression analysis. *p < 0.05, **p < 0.01, ***p < 0.001, ****p < 0.0001.

We further performed the analysis of immune cell infiltration in TCGA-KIRC cohorts to reveal critical associations between macrophage polarization and clinical prognosis (**Figure 7A**). Interestingly, the patients of high-risk group demonstrated a higher immune infiltration and lower stromal proportion compared to low-risk group (**Figure 7B**), indicative of a “hot” tumor microenvironment. Moreover, the patients in high-risk group had a higher score of overall macrophages and M1 subtypes while M2 subtype macrophages were not statistically significant (**Figure 7C**). As for M1/M2 ratio, high-risk group as well had a higher ratio compared to low-risk (**Figure 7C**). It was notable that Th2 cell score was dramatically enriched in high-risk group around 4.3 times (**Supplementary Table S1**). Patients with high M2 macrophage infiltration exhibited significantly better overall survival compared to those with low M2 levels (log-rank p = 0.018, **Figure 7D**). Conversely, a high M1 infiltration and M1/M2 macrophage ratio was strongly linked to improved survival (log-rank p < 0.01, **Figure 7D**), suggesting a harmful role of M1-polarized macrophages. Notably, total macrophage abundance alone showed no significant survival correlation (p=0.064, **Figure 7D**).

We further assess the clinical relevance of the newly identified macrophage subtype, PRDX1^+^LA-Mac. Patients with higher PRDX1^+^LA-Mac abundance exhibited significantly worse overall survival (log-rank p < 0.0001), with a more pronounced prognostic separation than observed for M1, M2, or total macrophages (**Figure 7E**). Spearman correlation analysis indicated only weak positive associations between PRDX1^+^LA-Mac and classical macrophage subsets (ρ = 0.11–0.18, all p < 0.05), suggesting that PRDX1^+^LA-Mac represents a transcriptionally and functionally distinct population (**Figure 7F**). Univariate Cox regression confirmed the strong prognostic value of PRDX1^+^LA-Mac, showing the highest hazard ratio and strongest statistical significance among all macrophage-related features (**Figure 7G**). Collectively, these results establish PRDX1^+^LA-Mac as a unique and highly informative prognostic macrophage subtype in KIRC.

### 3.8 Differential gene expression and pathway activation delineate high-risk KIRC subgroups

**Figure 8:**
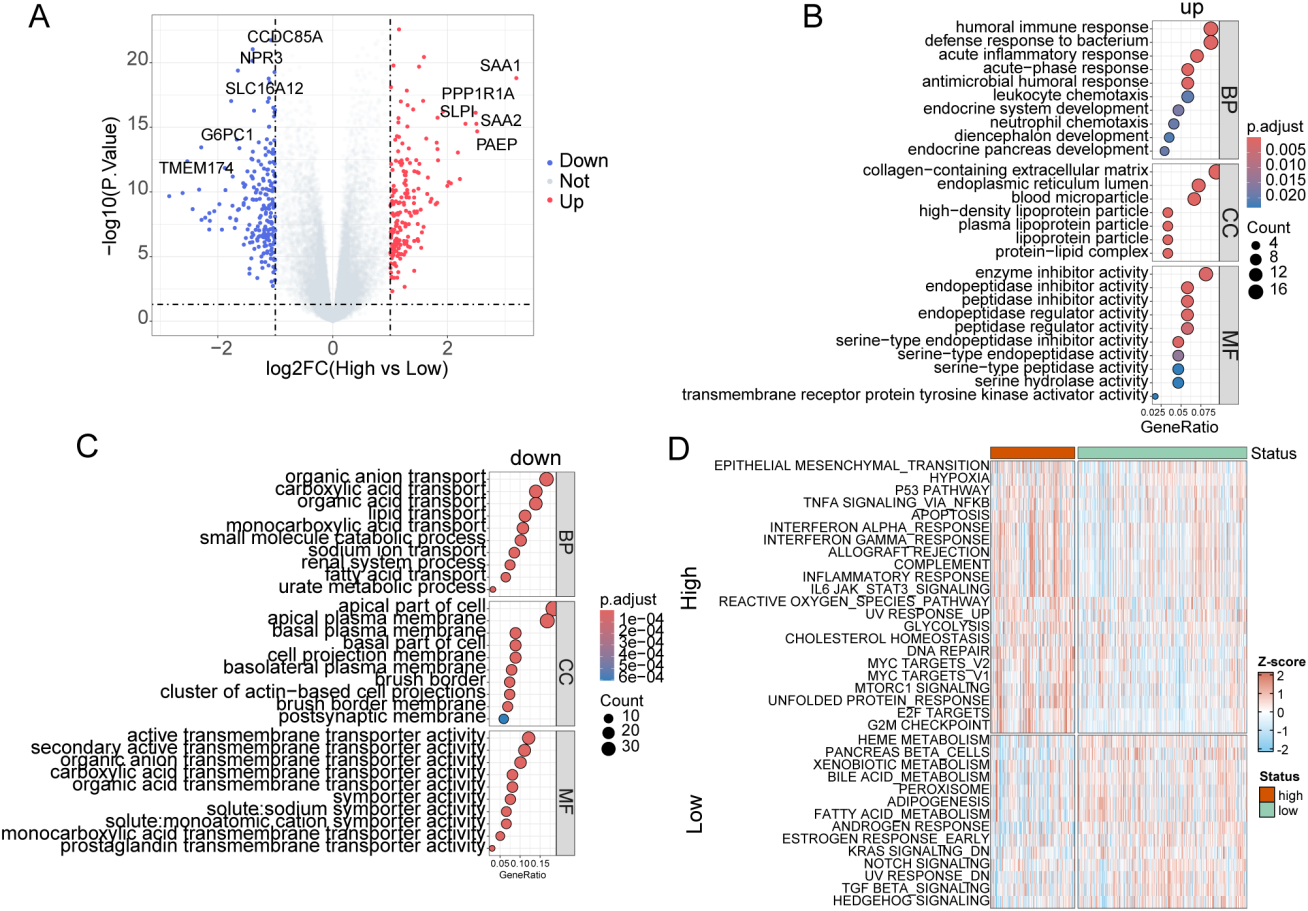
Transcriptomic and functional profiling of high-risk KIRC. **(A)** Volcano plot of DEGs (high *vs*. low risk), highlighting top upregulated (red) and downregulated (blue) genes. Dashed lines indicate significance thresholds (|log2FC| > 1, adj. p < 0.05). **(B, C)** GO enrichment analysis for upregulated **(B)** and downregulated **(C)** DEGs. Top terms for biological processes (BP), cellular components (CC), and molecular functions (MF) are labeled. **(D)** Heatmap of the hallmark gene set scores identified by ssGSEA between risk groups.

Transcriptomic analysis of TCGA-KIRC data identified 394 differentially expressed genes (DEGs) between high- and low-risk patients (|log2FC| > 1, adj. p < 0.05). Key upregulated genes in high-risk tumors included SAA1 (log2FC = 3.2, p.adj = 8.4e-17), PAEP (log2FC = 2.5, p.adj = 2.3e-13), and SLPI (log2FC= 2.3, p.adj = 7.3e-14), associated with inflammatory responses and immune evasion (**Figure 8A**). Downregulated genes such as NPR3 (log2FC = −1.6, p.adj= 3.0e-17), G6PC1 (log2FC = −2.3, p.adj= 2.7e-12) and SLC16A12 (log2FC = −1.77, p.adj=2.9e-17) were linked to metabolic dysregulation (**Figure 8A**). Gene Ontology (GO) enrichment revealed distinct functional profiles: upregulated DEGs were enriched in immune-related processes (acute inflammatory response, neutrophil chemotaxis, acute-phase response), lipid metabolism (high-density lipoprotein particle) and cellular matrix components (collagen-containing ECM), while downregulated DEGs implicated metabolic pathways (urate metabolism, fatty acid transport) and epithelial polarization (apical plasma membrane, brush border) (**Figures 8B, C**). Subsequent evaluation of hallmark signaling heterogeneity between high- and low-risk groups by ssGSEA revealed higher levels of proliferation, inflammatory response, and epithelial-mesenchymal transition in high-risk patients (**Figure 8D**). These findings highlight a pro-tumorigenic microenvironment in high-risk KIRC, characterized by immune activation, metabolic reprogramming, and stromal remodeling.

## 4 Discussion

In this comprehensive study, we employed single-cell RNA sequencing analysis to dissect the cellular heterogeneity of the kidney renal clear cell carcinoma (KIRC) tumor microenvironment, with a particular focus on macrophage polarization and its clinical implications. Through systematic profiling of 187,290 cells across normal kidney, tumor-adjacent, and tumor tissues, we identified five distinct macrophage subpopulations with unique functional states, revealing that tumor-associated macrophages deviate from the classical M1/M2 paradigm and exhibit complex mixed polarization patterns. Notably, we discovered that PRDX1+LA-Macs represent a key tumor-enriched subset associated with unfavorable prognosis. By integrating high-dimensional weighted gene co-expression network analysis (hdWGCNA) with machine learning approaches, we successfully developed a robust seven-gene prognostic signature (GPX1, LAIR1, FCGR1A, CD14, SERPING1, RGS1, and APOC1) derived from macrophage-specific gene modules, which demonstrated clinically meaningful predictive performance in both TCGA and CPTAC validation cohorts. Notably, high-risk patients defined by this signature exhibited distinct molecular features, including increased tumor mutation burden, heightened immune infiltration dominated by M1-like macrophages, and enrichment of oncogenic pathways related to inflammation, proliferation, and epithelial-mesenchymal transition (EMT). Collectively, our findings provide novel insights into the transcriptional complexity of TAMs in KIRC and establish a macrophage-derived prognostic model with translational potential, which may assist in patient risk stratification and identify potential therapeutic targets.

Among the identified five distinct macrophage subpopulations (FCN1+Inflam-Mac, PRDX1+LA-Mac, HERPUD1+LA-Mac, ALOX5AP+LA-Mac and OxP-Mac), OxP-Mac predominantly enriched in normal kidney tissues, whereas the other subtypes were mainly found in tumor regions. Notably, OxP-Mac exhibited the lowest expression of M1 and M2 polarization markers, representing a quiescent M0-like state. In contrast, the tumor-enriched PRDX1+ and HERPUD1+LA-Mac subtypes displayed mixed M1/M2 signatures, suggesting alternative activation states beyond the canonical M1/M2 classification. LA-Mac was the primary cellular source of hub genes compared to the other macrophage subsets and was previously reported to promote tumor cell EMT and immunosuppression (30). Among them, PRDX1+LA-Mac emerged as the most critical subpopulation, as indicated by its specificity in hub gene expression, deconvolution abundance, and ssGSEA scores. PRDX1, a member of the peroxiredoxin family, encodes antioxidant enzymes that protect cells from damage caused by reactive oxygen species (ROS). On one hand, PRDX1 promotes cholesterol efflux in macrophages, which may fuel tumor cell proliferation (39). On the other hand, it can alternatively activate macrophages into a pro-inflammatory phenotype, inducing cytokines such as IL-6, which are associated with cancer cell proliferation, drug resistance, and poor prognosis (40, 41). Pseudotime trajectory analysis further identified PRDX1+LA-Mac as an immune-infiltrated subset characterized by enhanced antigen presentation, negative regulation of immune responses, and disrupted immune coordination. This biased activation may play a pivotal role in shaping the TME, offering new insights into the cellular and functional imbalance within KIRC. These findings align with emerging evidence that TAMs exhibit functional plasticity and context-dependent roles within the TME.

Using hdWGCNA and cross-integration with TCGA, we identified seven macrophage-specific hub genes GPX1, LAIR1, FCGR1A, CD14, SERPING1, RGS1, and APOC1). FCGR1A encodes the high-affinity IgG receptor (FcγRI), mainly expressed on monocytes and macrophages (42). It plays a key role in immune responses by binding to the Fc segment of IgG antibodies, mediating functions like phagocytosis, antigen presentation, cytokine production, and immune complex clearance (43). It also has a key role in inflammatory responses (44). APOC1 is activated during monocyte differentiation into macrophages and is involved in lipid metabolism, particularly in high-density lipoprotein (HDL) and very low-density lipoprotein (VLDL) metabolism (45). It can inhibit cholesterol ester transfer protein (CETP) and impact cardiovascular disease by regulating lipoprotein metabolism (46). Additionally, APOC1 is overexpressed in various tumors, promoting cell proliferation, migration, and invasion (47). CD14 is a pattern recognition receptor that primarily acts as a co-receptor for Toll-like receptor 4 (TLR4). It identifies bacterial lipopolysaccharides (LPS), pathogen-associated molecular patterns (PAMPs), and damage-associated molecular patterns (DAMPs), thus activating innate immune responses (48). CD14 also helps clear apoptotic cells and regulates inflammatory and tolerance responses in barrier tissues like the skin and gastrointestinal tract (48). These genes not only played critical roles in macrophage differentiation but also correlated with clinical outcomes, highlighting their potential as therapeutic targets. The integration of transcriptional networks with functional polarization analysis provides a framework for understanding the molecular mechanisms underlying macrophage heterogeneity in KIRC.

We developed a prognostic random survival forest (RSF) model based on macrophage-associated hub genes, which demonstrated clinically meaningful predictive performance in both the training (TCGA) and validation (CPTAC) cohorts. Notably, FCGR1A, CD14, and APOC1 emerged as key features within the model, with APOC1 also serving as a prominent marker for tumor-enriched macrophage subtypes. These findings highlight the clinical relevance of macrophage-derived gene signatures for risk stratification and prognosis prediction in KIRC patients. However, we observed a substantial difference in AUC values between the training cohort (>0.9) and the validation cohort (~0.7). One plausible explanation is the underlying heterogeneity between the datasets, such as differences in overall survival time and mortality rates (49–52). Although these differences did not reach statistical significance in Kaplan–Meier analysis, they may still impact model performance. We also acknowledge the potential risk of overfitting in the current RSF model and recognize the need for further validation using additional independent datasets to confirm its generalizability and robustness.

Based on the RSF model, patients were stratified into high- and low-risk groups, and we systematically compared their mutation landscapes, immune infiltration profiles, molecular characteristics, and pathway alterations. High-risk patients exhibited higher TMB and specific gene (ZNF804B and FAM178A) mutation proportion, indicating these alterations may contribute to differential clinical outcomes. Additionally, immune cell infiltration analysis revealed higher immune infiltration in high-risk patients, particularly characterized by elevated Th2 and M1 macrophage scores. Th2 cells have been reported to be associated with poor prognosis and reduced responsiveness to PD-1 checkpoint blockade therapy (53, 54). However, other studies have demonstrated contrasting findings, suggesting context-dependent roles of Th2 cells and highlighting the heterogeneity of the TME (54). Moreover, recent mechanistic studies have provided additional insights into the pro-tumorigenic roles of M1 macrophages and Th2 cells in specific cancer contexts. M1 macrophages have been shown to induce PD-L1 expression in leader cells and promote collective invasion through TNF-α/CDK4/UPS14 signaling pathways. This finding supports our observation that M1 macrophage infiltration correlates with unfavorable prognosis and suggests a direct mechanistic link between M1 polarization and immune evasion (55). Similarly, therapeutic inhibition of Th2 cells has been demonstrated to enhance anti-tumor immunity and suppress tumor progression, as evidenced by studies showing that Saikosaponin A inhibits breast cancer by regulating Th1/Th2 balance (56). These mechanistic insights align with our findings of elevated Th2 scores in high-risk KIRC patients and provide biological rationale for the observed associations. Collectively, these inconsistencies with traditional paradigms underscore the urgent need for a more refined understanding of the molecular mechanisms underlying Th2 cell and macrophage function in cancer, particularly within the context of tissue-specific tumor microenvironments.

According to canonical M1/M2 gene signatures and immune infiltration estimates based on the ssGSEA method, we observed results that contradict traditional assumptions. The classical paradigm posits that M1 macrophages exert anti-tumor effects while M2 macrophages promote tumor progression. In our study, M1 macrophage infiltration was associated with unfavorable prognosis, whereas M2 infiltration correlated with better outcomes. Notably, a higher M1/M2 ratio was significantly linked to poorer survival. M1 macrophages are known for their hyperinflammatory phenotype, which can be exploited by cancer cells to support proliferation (57). Previous studies have shown that tumor cells can induce M1-like polarization through lncRNA-mediated mechanisms, thereby enhancing pro-tumor inflammatory responses (58). Consistently, we found that high-risk patients were enriched in inflammation-related pathways. In contrast, M2 macrophages have also been implicated in suppressing metastasis by promoting vascular normalization and stabilization (59). These observations highlight the context-dependent roles of macrophages and the limitations of the traditional M1/M2 framework in fully capturing the diverse functional states and plasticity of TAMs.

Building upon this, our study further demonstrates that the PRDX1^+^LA-Mac subtype represents a highly tumor-promoting macrophage population in KIRC. Deconvolution-based analysis revealed that higher PRDX1^+^LA-Mac abundance was strongly associated with poorer overall survival, exceeding the prognostic impact of classical M1, M2, or total macrophage infiltration. Spearman correlation analysis indicated only weak associations with M1 and M2 macrophages (ρ = 0.11–0.18), suggesting that PRDX1^+^LA-Mac represents a transcriptionally and functionally distinct population rather than a simple combination of canonical M1/M2 states. Univariate Cox regression further confirmed its superior predictive value, with the highest hazard ratio among all macrophage-related features. Interestingly, PRDX1^+^LA-Mac exhibits a hybrid transcriptional profile, encompassing both M1- and M2-related gene programs, which may underlie its strong pro-tumorigenic activity. These results emphasize that the prognostic relevance of TAMs in KIRC cannot be fully captured by traditional polarization markers alone and underscore the importance of identifying and characterizing distinct macrophage subpopulations for understanding tumor-immune interactions. Future studies should aim to dissect the dynamic reprogramming of macrophage phenotypes during disease progression and therapy, supported by experimental validation.

Our study provides a comprehensive characterization of macrophage subpopulations and gene networks in KIRC, revealing their diagnostic and therapeutic potential. The identification of macrophage-derived prognostic signatures and their associations with clinical outcomes highlights the potential of targeting macrophage polarization in KIRC treatment. Future studies should validate these findings in independent cohorts and investigate the functional roles of the identified hub genes using preclinical models. Moreover, integrating additional independent datasets—particularly at the single-cell level—will offer a more complete understanding of the macrophage landscape in KIRC. Finally, as this study is based on multi-omics integrative analyses, experimental validation is needed to elucidate the mechanisms by which these hub genes regulate macrophage polarization and contribute to KIRC progression.

## 5 Conclusion

This multi-omics study reveals the transcriptional and functional heterogeneity of tumor-associated macrophages (TAMs) in KIRC, uncovering distinct macrophage subtypes with prognostic relevance. By integrating single-cell and bulk transcriptomic data, we establish a robust macrophage-derived prognostic signature with strong translational potential. Our findings highlight the roles of macrophage polarization in KIRC progression, offering novel molecular targets for prognosis prediction, risk stratification, and therapeutic intervention in KIRC.

## Data Availability

The original contributions presented in the study are included in the article/**Supplementary Material**. Further inquiries can be directed to the corresponding author.

## Supplementary material

Supplementary Figure 1 Survival distribution heterogeneity between the TCGA and CPTAC KIRC cohorts. **(A)** Violin plot comparing overall survival time between the TCGA- and CPTAC KIRC cohorts. **(B)** Heatmap displaying the distribution of survival status across the two cohorts. **(C)** Kaplan–Meier survival curves comparing overall survival between TCGA and CPTAC KIRC cohorts.

Supplementary Figure 2 PRDX1+LA-Mac and RSF feature genes are enriched in high-risk KIRC patients and correlate with poor prognosis. **(A, B)** MuSiC deconvolution analysis of macrophage subtype abundances in TCGA **(A)** and CPTAC **(B)** bulk transcriptomic data. **(C, D)** ssGSEA scores based on the top 30 marker genes of macrophage subtypes in TCGA **(C)** and CPTAC **(D)** cohorts. **(E, F)** ssGSEA scores of the top 10 hub genes from the macrophage-specific module in TCGA **(E)** and CPTAC **(F)** cohorts.
