# Supplementary figures and images for: Integrated multi-omic profiling reveals macrophage-driven prognostic signatures in clear cell renal cell carcinoma through machine learning optimization
Source: Front Immunol. 2026 Jan 12;16:1612262. doi: 10.3389/fimmu.2025.1612262 (PMC12833068; doi:10.3389/fimmu.2025.1612262)

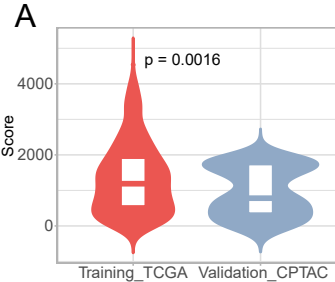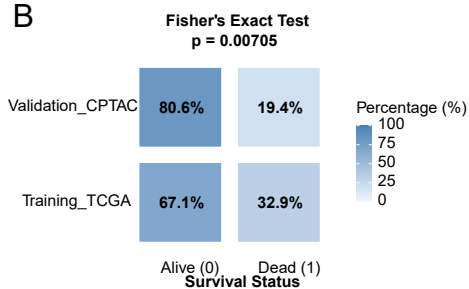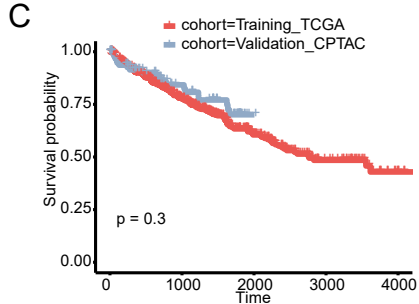

Supplement: Supplementary Figure 1 — Survival distribution heterogeneity between the TCGA and CPTAC KIRC cohorts. (A) Violin plot comparing overall survival time between the TCGA- and CPTAC KIRC cohorts. (B) Heatmap displaying the distribution of survival status across the two cohorts. (C) Kaplan–Meier survival curves comparing overall survival between TCGA and CPTAC KIRC cohorts. [file Image1.pdf]

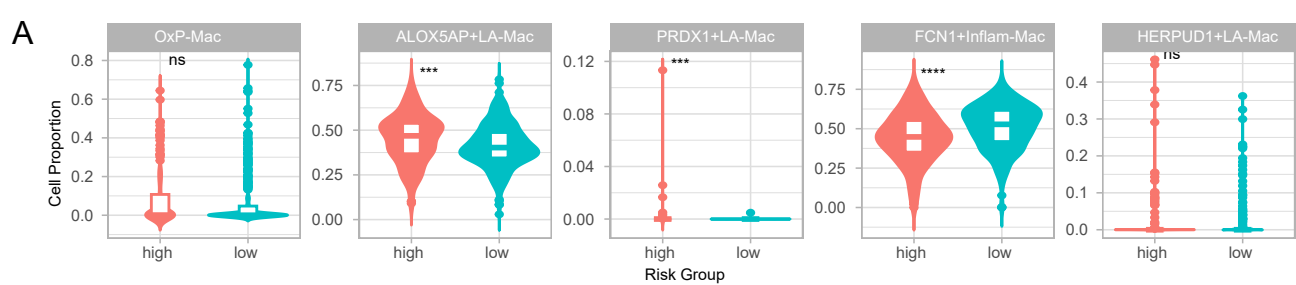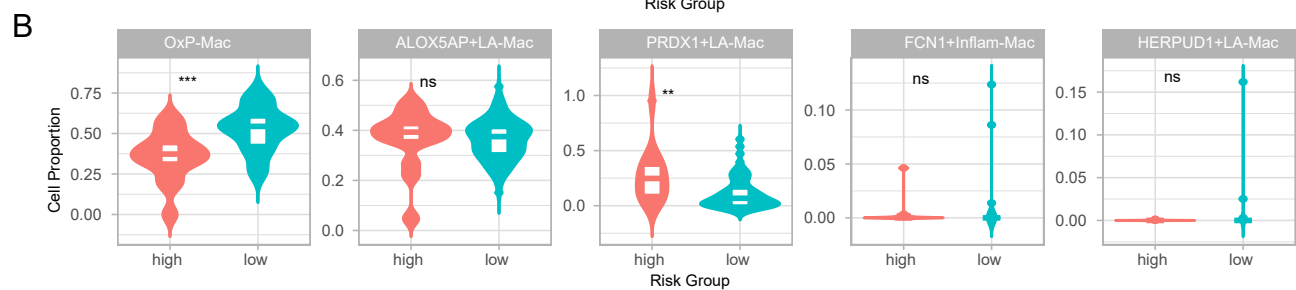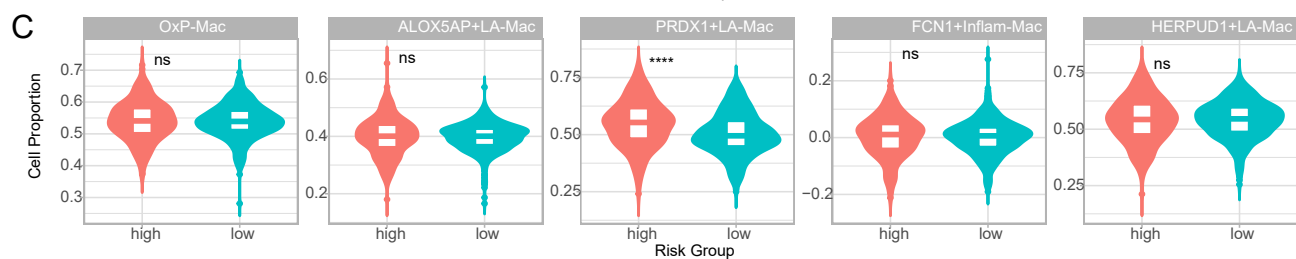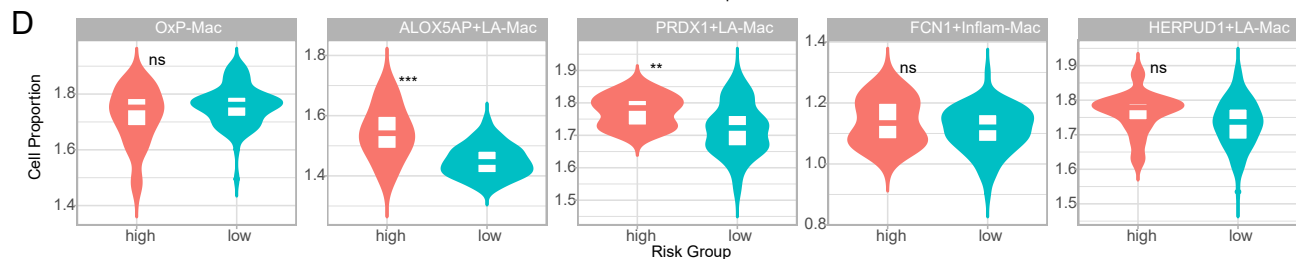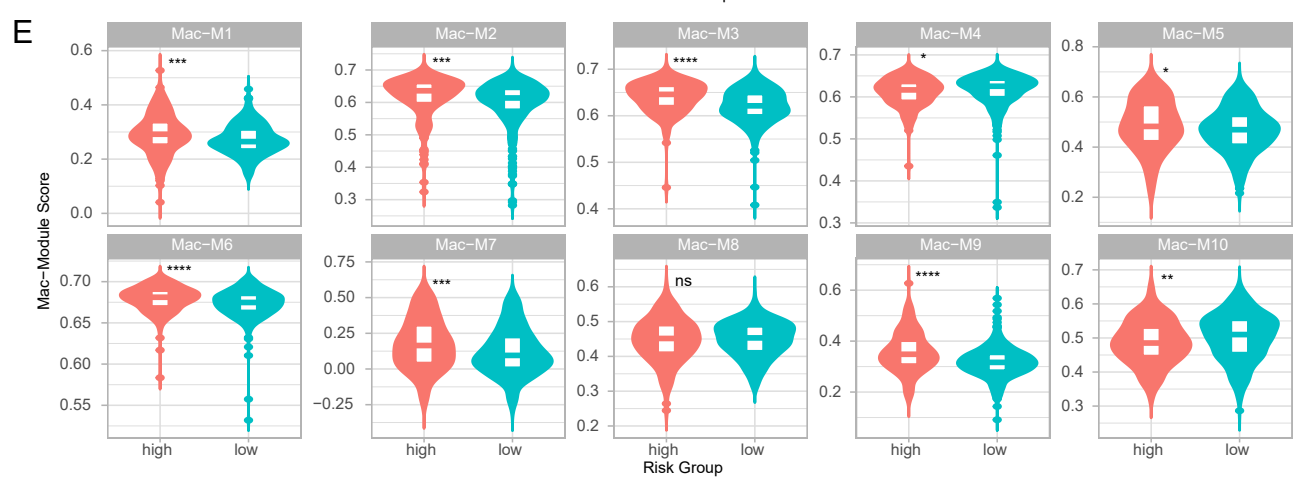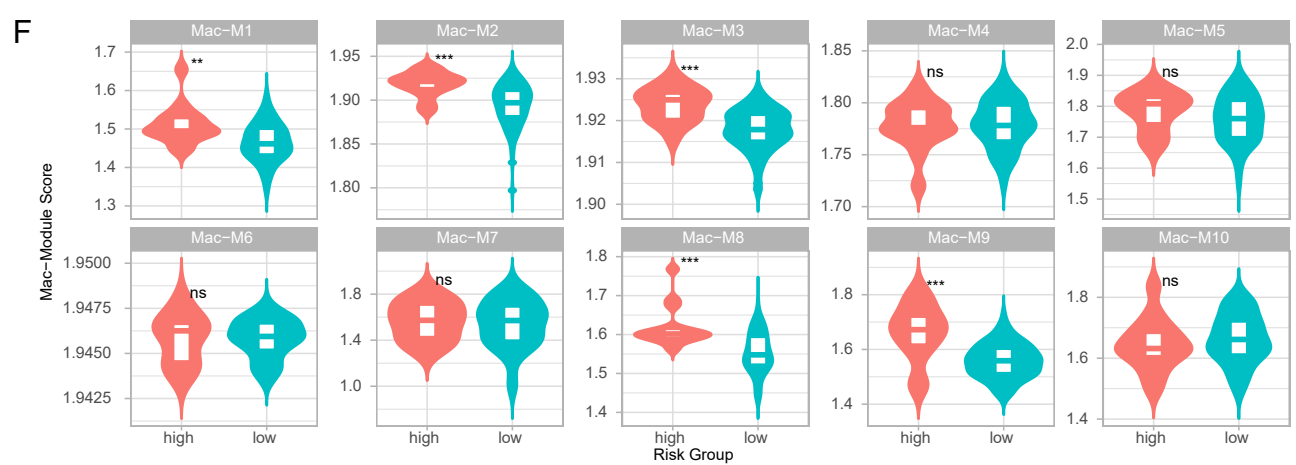

Supplement: Supplementary Figure 2 — PRDX1+LA-Mac and RSF feature genes are enriched in high-risk KIRC patients and correlate with poor prognosis. (A, B) MuSiC deconvolution analysis of macrophage subtype abundances in TCGA (A) and CPTAC (B) bulk transcriptomic data. (C, D) ssGSEA scores based on the top 30 marker genes of macrophage subtypes in TCGA (C) and CPTAC (D) cohorts. (E, F) ssGSEA scores of the top 10 hub genes from the macrophage-specific module in TCGA (E) and CPTAC (F) cohorts. [file Image2.pdf]
